# Knockdown of ribosomal protein S6 suppresses proliferation, migration, and invasion in epithelial ovarian cancer

**DOI:** 10.1186/s13048-020-00707-7

**Published:** 2020-08-31

**Authors:** Xueqing Yang, Luxi Xu, Yu-e Yang, Chang Xiong, Jinjin Yu, Yuan Wang, Yaying Lin

**Affiliations:** 1 Department of Medicine, Jiangnan University, Wuxi, 214000 People’s Republic of China; 2 Department of Obstetrics and Gynecology, Affiliated Hospital of Jiangnan University, Wuxi, 214000 People’s Republic of China

**Keywords:** Ribosomal protein S6 (RPS6), Epithelial ovarian cancer (EOC), G0G1 arrest, Cell proliferation, Invasion, Migration

## Abstract

**Background:**

Ovarian cancer typically is diagnosed late because insensitivity and lack of specificity of current biomarkers prior to its clinical detection. Ribosomal protein S6 (RPS6) is a ribosomal protein involved in the ribosomal 40S subunit, but its biological role in epithelial ovarian cancer (EOC) is still unknown.

**Results:**

RPS6 was elevated in EOC compared to normal ovarian tissues and adenomas. Higher expression of RPS6 predicted worse prognosis in EOC. The level of RPS6 was correlated with clinical stage, histological type and pathological grade. Knockdown of RPS6 reduced the proliferation of ovarian cancer cell lines SKOV-3 and HO8910, and inhibit the migration and invasion ability. It revealed that cells arrested at G0G1 phase after knockdown of RPS6, and the expressions of CyclinD1, Cyclin E, CDK2, CDK4, CDK6 and pRb were also reduced.

**Conclusions:**

RPS6 is involved in EOC and knockdown of RPS6 could inhibit the proliferation, invasion and migration ability of EOC in vitro by inducing G0/G1 phase arrest. RPS6 is expected to be a novel biomarker and molecular target to the EOC.

## Background

Ovarian cancer is the seventh most commonly diagnosed cancer and the fifth leading cause of cancer-related death among women [1, 2]. A major factor implicated in the high mortality rates from ovarian cancer is that the disease has frequently reached an advanced stage at primary diagnosis [3], as there are no specific clinical symptoms in the early stages. Besides, late detection in part is related to findings since risk-reducing surgeries in BRCA mutation carriers: some of the cancers diagnosed as ovarian cancer may actually arise in the fimbria of the Fallopian Tubes, thus rarely being diagnosed in stage I [4, 5]. Consequently, 70% of patients have advanced disease at primary diagnosis, for which the 5-year survival rate is approximately 29%, compared with 92% for early-stage disease [6].

In eukaryotes, mature ribosomes (80S) contain four RNAs and roughly 80 proteins, which together make up the 60S large and 40S small ribosomal subunits [7]. The interaction between mRNA and the small 40S subunit is a key process in protein synthesis. Ribosomal proteins also have other roles, including participating in DNA repair, and regulating cell proliferation, apoptosis, and differentiation [8]. RPS6 is an important protein constituent of the 40S small subunit, located in the mRNA binding site of cytosolic ribosomes, and is the first ribosome protein discovered to undergo phosphorylation [9]. RPS6 has important functions in tumorigenesis and development. Abnormal expression of RPS6 or phosphorylated-RPS6 has been detected in vulvar squamous cell carcinoma [10], cervical cancer [11], oral squamous cell carcinoma [12], non-small cell lung cancer [13], renal cell carcinoma [14], esophageal squamous cell carcinoma [15]. In addition, RPS6 has a crucial role in the mechanisms of action of antitumor drugs. The sensitivity of human epidermal growth factor receptor (HER)-2-positive breast cancer to trastuzumab is increased by miR-129-5p via downregulation of RPS6 [16]. Further, dephosphorylation of RPS6 can inhibit the mechanistic target of rapamycin (mTOR) pathway in tumors, inhibiting their growth and metastasis [17]. Nevertheless, the specific function of RPS6 in the development of ovarian cancer remains unclear and warrants further investigation.

In this study, we evaluated RPS6 expression in EOC and normal tissues, and analyzed corresponding clinical data. The role of RPS6 in ovarian cancer was also assessed by silencing its expression in ovarian cancer cell lines and conducting functional assays.

## Material and methods

### Patients and tissue samples

Total of 183 EOC specimens were obtained from primary debulking surgeries (PDS) performed in the Gynecology Department at the Affiliated Hospital of Jiangnan University, from January 2014 to December 2018; residual tumors were all < 1 cm. Surgical staging was conducted according to the FIGO system, and histological type and pathology grade were determined according to the WHO classification. All patients were followed-up according to the NCCN guidelines. Benign ovarian adenomas (*n* = 37) were obtained by simple ovarian surgery, and normal ovary tissue samples (*n* = 32) were obtained from hysterectomies for benign uterine diseases.

### Immunohistochemistry

All tissue samples were fixed in 10% neutral buffered formalin, embedded in paraffin, sectioned at 4 μm. Immunohistochemistry procedures were performed as previously described [18]. Tissue samples were then incubated with anti-RPS6 primary antibody (Abcam, ab70227, diluted 1:100) and observed under an optical microscope. Immunohistochemical staining was assessed independently by two experienced, senior pathologists, who scored them as follows: 0, no staining; 1, light yellow; 2, yellow; and 3, brown. The percentage of positive cells was scored using a semi-quantitative method, as follows: 0, < 10%; 1, 10–25%; 2, 26–50%; 3, 51–75%; and 4, > 75%. RPS6 expression was classified according to the sum of these two scores, as follows: ≤ 3, low expression and > 3, high expression.

### Ovarian cancer cell lines and cell culture

The human ovarian cancer cell line, SKOV3, was purchased from American Type Culture Collection, and cultured in McCoy’s 5A medium containing 10% fetal bovine serum, 100 μg/ml penicillin, and 100 μg/ml streptomycin. While HO-8910 cells were obtained from the Cell Bank of the Chinese Academy of Sciences, and cultured in Roswell Park Memorial Institute (RPMI) 1640 medium containing 10% fetal bovine serum, 100 μg/ml penicillin, and 100 μg/ml streptomycin. Cells were incubated in a 37 °C, 5% CO_2_, saturated humidity incubator.

### Construction and transfection of lentiviral Short Hairpin RNA (shRNA)

A lentiviral packaging plasmid pFH-L including a green fluorescent protein (GFP) tag was purchased from Gene Pharma (COA-020, Suzhou, China). The two shRNA sequences targeting the human *RPS6* gene (NM_001010.3) and the negative control (NC) sequence were as follows: 5′-GAAGCAGCGTACCAAGAAA-3′ (shRPS6(S1)), 5′-GGAACAAATTGCGAAGAGA-3′ (shRPS6(S2)), and 5′-TTCTCCGAACGTGTCACGT-3′ (shCon), respectively. When the cells reached 30% confluence in six-well plates, lentiviral solution was added with a final concentration of 5 μg/ml Polybrene. Cells were transduced using the lentiviral constructs, and the medium was replaced after 24 h of culture. After 72 h, the GFP fluorescence level from the lentiviral vector was observed under a fluorescence microscope. Five days after transfection, positively expressing cells were selected in medium containing 2 μg/ml puromycin in order to obtain stably transfected cell lines. Finally, real-time quantitative (qRT)-PCR and western blotting were conducted to verify RPS6 knockdown.

### qRT-PCR

Total RNA was extracted from cells collected from six-well plates by adding Trizol reagent (Vazyme Biotech, Nanjing, China), and quantified using a Nanodrop 2000 spectrophotometer (Thermo Fisher Scientific, MA, USA). PrimeScript™ RT Master Mix Kit (RR036A, Takara Bio, Tokyo, Japan) was used for reverse transcription, and a TB Green™ Premix Ex Taq™ Kit (RR820A, Takara Bio, Tokyo, Japan) was used for PCR amplification. The PCR reactions were conducted in 20-μl reaction mixtures containing. PCR amplification was conducted as follows: pre-denaturation at 95 °C 30 s, then 40 cycles of denaturation at 95 °C for 5 s, annealing at 60 °C for 34 s, and extension at 60 °C for 60 s. qRT-PCR was conducted using an Applied Biosystems ViiA 7 Dx instrument (Life Technologies Inc., Gaithersburg, MD, USA). Primer sequences were: *RPS6* forward, 5′-TGTTACTCCACGTGTCCTGC-3′ and reverse, 5′- AAGTCTGCGTCTCTTCGCAA-3′; and β-actin forward, 5′-GTGGACATCCGCAAAGAC-3′ and reverse, 5′-AAAGGGTGTAACGCAACTA-3′. Gene expression levels were determined using the 2^-ΔΔCt^ method, with β-actin as an internal reference.

### Western blot analysis

Total proteins were extracted from cells collected by adding RIPA Lysis and Extraction Buffer (Thermo Fisher Scientific, Waltham, MA, USA) plus Protease Inhibitor Cocktail (ComWin Biotech, Beijing, China) and incubating on ice for 30 min at 4 °C. Protein extracts were quantified using a Bicinchoninic Acid Protein Assay Kit (Thermo Fisher Scientific, Waltham, MA, USA). 26 μg total protein was added per well to acrylamide gels and then separated by SDS-PAGE and transferred to PVDF membranes. Next, the membranes were blocked using 5% skim milk or, for detection of phosphorylated proteins, with 5% bovine serum albumin, for 1 h at room temperature. Thereafter, the membranes were incubated overnight at 4 °C with primary antibodies, including: anti-RPS6 (1:1000, ab40820, Abcam, Cambridge, UK), anti-CDK2 (1:1000, #2546, CST, Boston, MA, USA), anti-CDK4 (1:1000, #12790, CST), anti-CDK6 (1:1000, #13331, CST), anti-Cyclin D1 (1:1000, #2978, CST), anti-Cyclin E (1:1000, #4129, CST), anti-retinoblastoma (Rb; 1:1000, #9313, CST), anti-phosphorylated retinoblastoma (pRb; 1:1000, #8516, CST), and anti-β-actin (1:1000, AF0003, Beyotime, Shanghai, China). The following day, the membranes were incubated with secondary antibody (1:1000, A0216 and A0208, Beyotime) for 1 h at room temperature and washed three times with tris-buffered saline with Tween 20 (TBST) for 15 min each time. Finally, a hypersensitive enhanced chemiluminescence (ECL) kit (Beyotime) was used to detect specific protein bands, using a Bio-Rad ChemiDoc XRS Imaging System (Bio-rad Laboratories Inc., California, USA) for visualization and quantification; grayscale intensity values of protein bands were analyzed using Image J software (National Institutes of Health, Bethesda, MD, USA), with β-actin as the internal reference.

### Cell proliferation assay

Cell proliferation ability was monitored using a Cell Counting Kit-8 (CCK-8, Dojindo, Japan). SKOV-3 and HO-8910 cells in the log phase of growth were plated in 96-well plates at a density of 3000 cells/well with 100 μl of medium. Thereafter, 10 μl CCK-8 solution was added at 8, 24, 48, 72, 96, or 120 h, followed by incubation at 37 °C for 2 h in the dark. Finally, optical density (OD) at 450 nm was measured using a microplate reader (Hidex, Finland). At least three independent experiments were performed.

### Colony formation assay

SKOV3 cells in the RPS6 knockdown and LV-shCon groups were inoculated in six-well plates at 1200 cells per well, while HO-8910 cells were added at 1500 cells per well. After 10 days of growth, the cultures were terminated. Cells were fixed in 4% paraformaldehyde for 15 min and then stained with 0.1% crystal violet for 30 min. The number of cell colonies in each group was counted under a microscope, and the percentage of colonies, relative to the number of cells inoculated, was used to calculate the colony formation rate for each group.

### Cell cycle assays

Ovarian cancer cells in the logarithmic growth phase were inoculated into six-well plates (1 × 10^6^ cells/well), cultured for 48 h, digested with trypsin, and fixed in pre-chilled 70% alcohol at 4 °C for 12 h. The cells were then resuspended in 400 μl propidium iodide staining solution (staining buffer: 25× propidium iodide: RNase A, 400:15:1) and incubated at room temperature for 30 min in the dark. Changes in the cell cycle were then monitored by flow cytometry (Becton Dickinson, San Diego, CA, USA). The data were analyzed using Modfit LT 3.0 software (Verity Software House, Topsham, ME, USA) and the proportion of cells in each phase of the cell cycle was determined.

### Scratch assay

Transfected SKOV3 and HO-8910 cells were seeded in six-well plates. When the cells reached 90% confluence, a 200-μl sterile pipette tip was used to make a vertical wound in each dish. The cells were then washed gently with PBS, and serum-free medium was added to each well for culture. The scratched area was observed under an inverted microscope at 0, 24, and 48 h, and images of the wound were acquired. Migration ability is directly proportional to the scratch healing rate.

### Transwell assay

Transwell membranes were obtained from Corning Inc. (NY, USA), and the test was performed according to the manufacturer’s protocol. Cells (SKOV3 at 1 × 10^6^ cells/150 μl and HO-8910 at 1.5 × 10^6^ cells/150 μl) in serum-free medium were added to the upper transwell chambers, while 500 μl culture medium supplemented with 20% serum was placed in the lower chamber. After cultured for 24 h, the cells were fixed with 4% paraformaldehyde for 30 min and stained with 0.1% crystal violet staining solution for 15 min. The number of cells on the lower layer of the microporous membrane was observed using an inverted microscope. Five fields were randomly selected for each membrane in order to observe the number of cells and calculate the mean value, using Image J software (National Institutes of Health, Bethesda, MD, USA) for statistical analysis. The number of invading cells indicated the invasive capacity. The cell migration assay was similar to the invasion assay, except that Matrigel was not required and the number of inoculated cells differed (SKOV3 at 5 × 10^6^ cells/150 μl and HO-8910 at 8 × 10^6^ cells/150 μl).

### Statistical analysis

SPSS 24.0 software (IBM, Armonk, NY, USA) and GraphPad Prism 8.0 (GraphPad Software, San Diego, CA, USA) were used for statistical analyses and drawing figures. All survival end-points were calculated starting from the PDS date. OS and PFS were analyzed using Kaplan-Meier curves applying log-rank tests (Mantel-Cox). Comparisons between groups were performed using the Student’s t test for continuous variables and the chi-square (χ^2^) test for categorical variables. Each experiment was performed at least three times and *p* < 0.05 was considered statistically significant.

## Results

### RPS6 is overexpressed in EOC tissues and its expression is associated with poor clinical outcomes

**Fig. 1:**
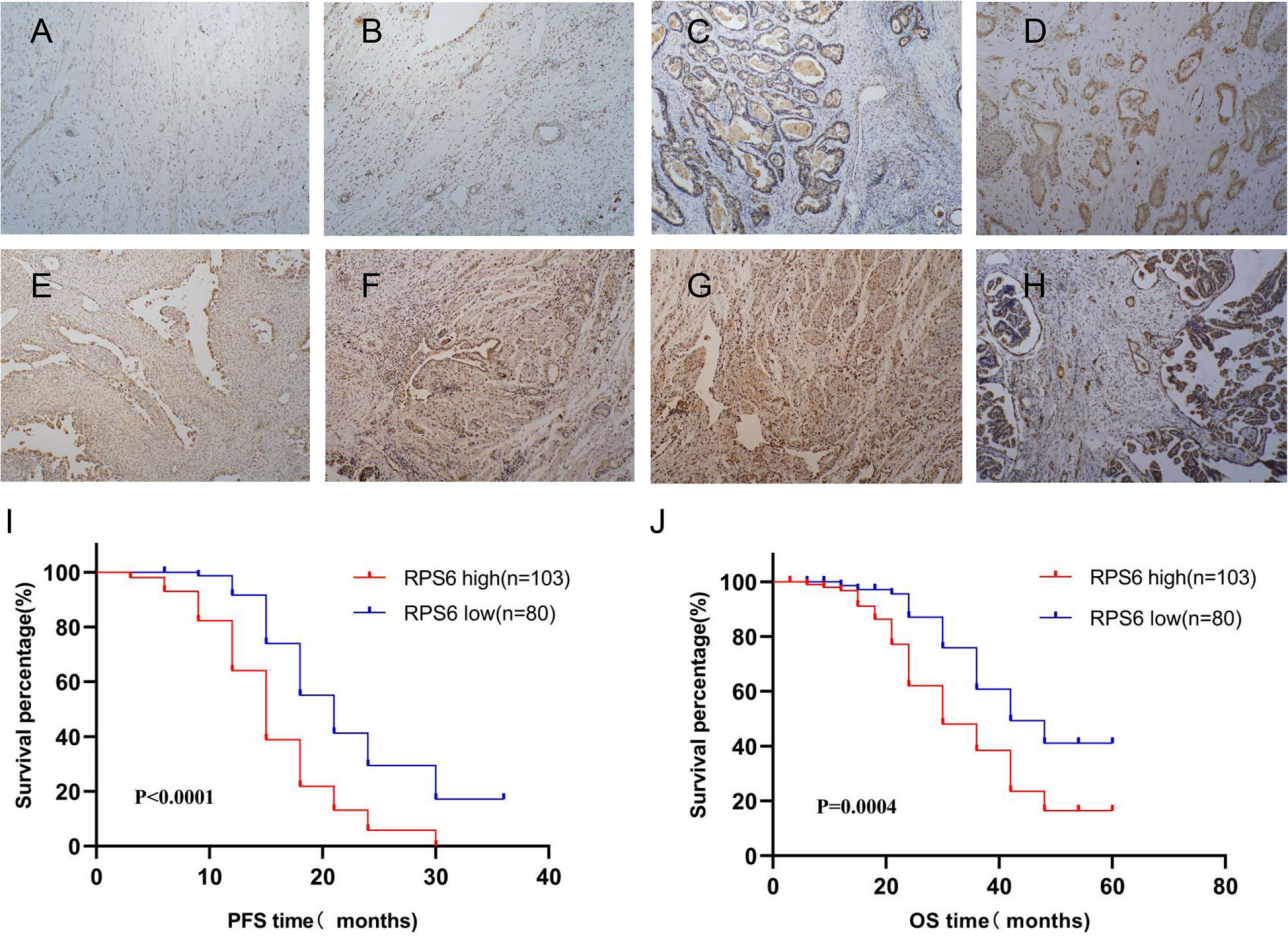
RPS6 is upregulated in EOC tissues. Images showing IHC staining of EOC, adenoma, and normal ovarian tissue samples. **a** Negative staining for RPS6 in normal tissue. **b** Weak staining for RPS6 in adenoma tissue. **c** Strong staining of RPS6 in ovarian clear cell carcinoma tissue. **d** Strong staining of RPS6 in serous adenocarcinoma. **e–h** RPS6 expression in FIGO stage I, II, III, and IV EOC tissues, respectively. PFS **(i)** and OS **(j)** of patients with EOC. Original magnification, × 100

RPS6 expression was examined by immunohistochemistry in a total of 252 tissue samples, of which 183 were EOC. RPS6 was mainly distributed in the nucleus of ovarian tumor cells (Fig. 1a-h). Using the scoring system described above, the samples were divided into two groups based on high and low RPS6 expression. The RPS6 protein level was lower in normal ovarian tissues (Fig. 1a) than in adenomas (Fig. 1b), and levels in adenomas and normal ovarian tissue were lower than those in EOC (Fig. 1c, d), and the differences were significant (Table 1). There was no significant difference in RPS6 expression between serous and non-serous carcinomas (Fig. 1c, d; Table 1), nor between samples from individuals aged > or ≤ 50 years; however, RPS6 expression was significantly associated with FIGO stage (Fig. 1e-h), and pathological grade (Table 1).

**Table 1:** Association of RPS6 expression with ovarian tissue features

Features	Number of patients	Expression (n, %)		*P*
		Low	High	
Tissue type				<0.001
normal	32	27 (84.4)	5 (15.6)	
adenomas	37	24 (64.9)	13 (35.1)	
EOC	183	80 (43.7)	103 (56.3)	
Age				NS
< 50	47	19 (40.4)	28 (59.6)	
⩾50	136	61 (44.9)	75 (55.1)	
FIGO stage				0.011
I	14	10 (71.4)	4 (28.6)	
II	29	18 (62.1)	11 (37.9)	
III	107	41 (38.3)	66 (61.7)	
IV	33	11 (33.3)	22 (66.7)	
Histological type				NS
Serous	135	57 (42.2)	78 (57.8)	
Non-serous	48	23 (47.9)	25 (52.1)	
Pathology grade				0.038
Low	67	36 (53.7)	31 (46.3)	
High	116	44 (37.9)	72 (62.1)	

Patients with high RPS6 expression had worse overall survival relative to those with low expression (median OS, 30 vs. 42 months) (Fig. 1j). Furthermore, high RPS6 expression was associated with poor progression-free survival, with median PFS values of 15 vs. 21 months in patients with high vs. low RPS6 levels (Fig. 1i). Hence, high RPS6 expression predicted significantly inferior outcomes, and was associated with increased risks of death and disease progression.

### RPS6 was significantly downregulated by shRNA lentivirus in ovarian cancer cell lines

**Fig. 2:**
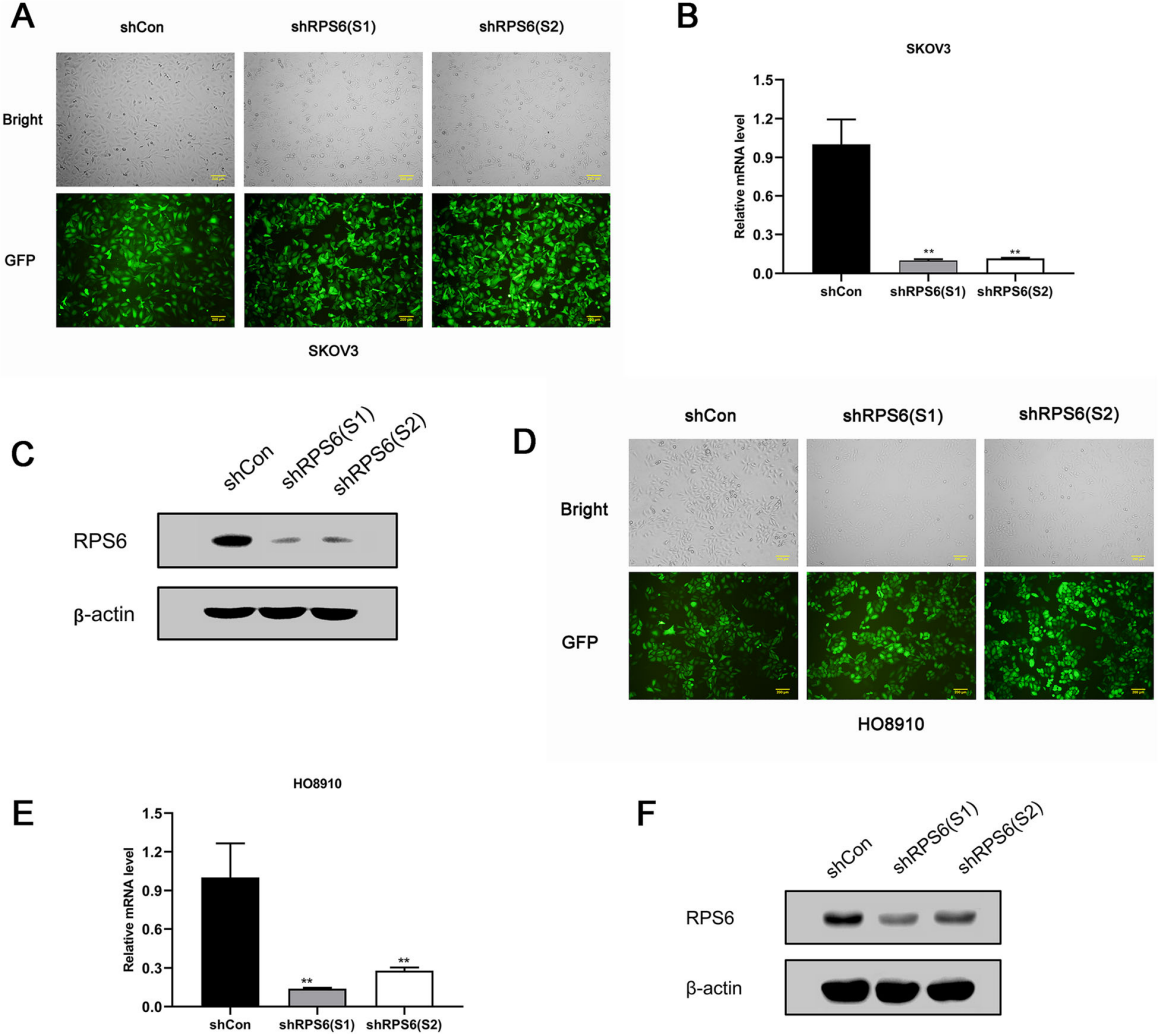
Knockdown of RPS6 in SKOV3 and HO-8910 cells using LV-shRNA. Lentivirus transduction in SKOV3 and HO-8910 cells was detected by fluorescence microscopy at a magnification of 100× (lower); scale bar: 200 μm. These results suggest that both SKOV3 and HO-8910 cells had high levels of GFP expression **(a, d)**. Depletion of *RPS6* mRNA in SKOV3 and HO-8910 cells lines treated with RPS6-shRPS6 was determined by qRT-PCR **(b, e)**. Decreased expression of RPS6 protein was detected by western blotting after transfection **(c, f)**

Lentiviral vector-mediated transfection was used to determine the effect of RPS6 silencing in ovarian cancer cells. After transfection (72 h), the number of cells expressing GFP was observed using a fluorescence microscope and the infection efficiency was found to be > 90% in both SKOV3 and HO-8910 cells (Fig. 2a, d). Subsequently, the knockdown efficiency of RPS6 was determined by qRT-PCR. *RPS6* mRNA levels in SKOV3 and HO-8910 cells were drastically reduced by treatment with both LV-shRPS6(S1) and LV-shRPS6(S2) compared with LV-shCon (Fig. 2b, e). RPS6 protein expression was evaluated by western blot assay, and the results showed that levels were markedly reduced by LV-shRNAs in both SKOV3 and HO-8910 cells (Fig. 2c, f).

### Knockdown of RPS6 inhibits SKOV3 and HO-8910 cell proliferation in vitro

**Fig. 3:**
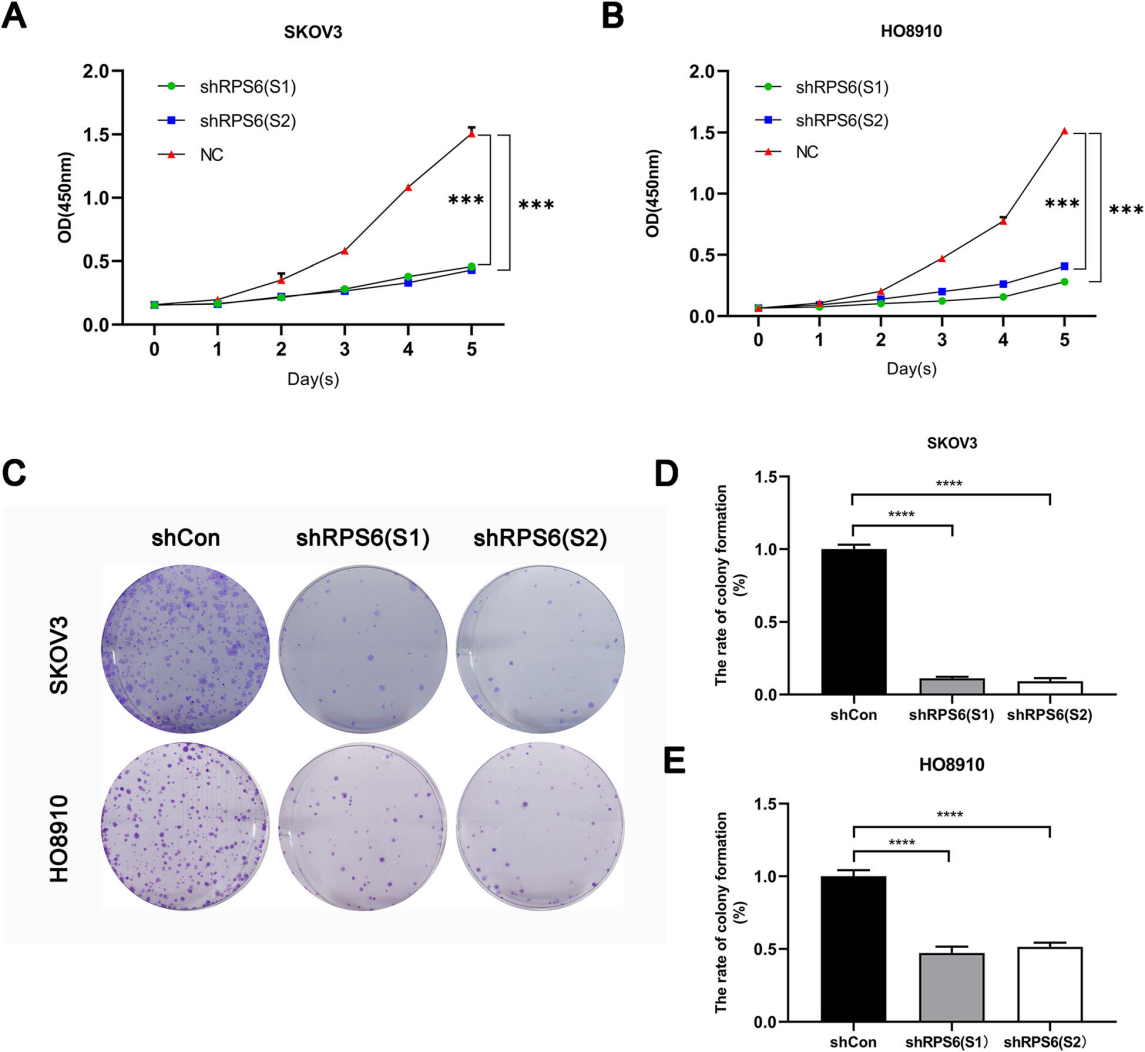
RPS6 knockdown inhibited cell proliferation and colony formation. Results of CCK-8 proliferation assay showing the inhibition of proliferation of both SKOV3 **(a)** and HO-8910 **(b)** cells following treatment with LV-shRPS6 at 8, 24, 48, 72, 96, and 120 h. RPS6 silencing reduced colony formation by ovarian cancer cells **(c–e)**. All comparisons are with the LV-shCon group. ***p < 0.001, ****p < 0.0001

The functional effect of RPS6 knockdown on SKOV3 and HO-8910 cell proliferation was evaluated by CCK-8 and colony formation assays. The results demonstrated that downregulation of RPS6 by LV-shRNA remarkably impeded the viability of SKOV3 and HO-8910 cells, relative to the LV-shCon group, in a time-dependent manner (Fig. 3a, b). Simultaneously, colony formation assays showed that knockdown of RPS6 led to a significant decrease in the number of colonies of SKOV3 and HO-8910 cells, by approximately 90 and 50%, respectively, compared with the LV-shCon group (Fig. 3c-e).

### Repression of RPS6 inhibits EOC cell migration and invasion in vitro

**Fig. 4:**
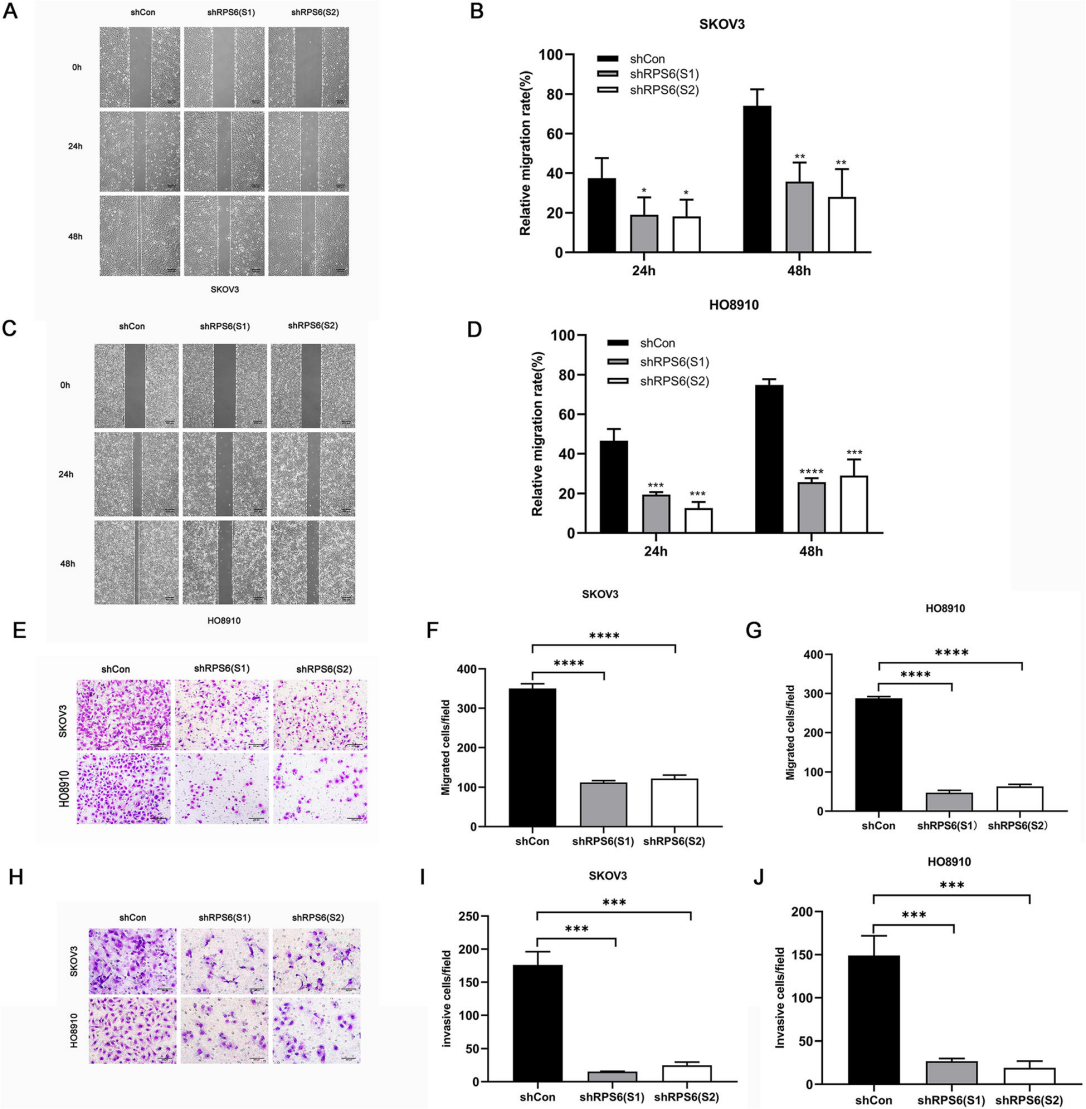
Knockdown of RPS6 inhibited ovarian cancer cell migration and invasion in vitro. Migration ability was assessed using the scratch test after 24 and 48 h (magnification 40×; scale bar: 500 μm) on SKOV3 **(a, b)** and HO-8910 **(c, d)** cells. Transwell migration assays were conducted to assess the effect of RPS6 knockdown on SKOV3 and HO-8910 cell migration (magnification 100×; scale bar: 200 μm) **(e)**. Graph of transwell migration assay data for SKOV3 cells with RPS6 knocked down (112 ± 4.90 and 122 ± 8.38), showing reduced cell migration, compared with control cells (shCon) (350 ± 12.25) (*N* = 3, 2-tailed unpaired t-test, *p* < 0.0001) **(f)**. Graph of transwell migration assay data for HO-8910 cells with RPS6 knocked down (47 ± 5.73 and 63 ± 5.25), showing reduced cell migration, compared with shCon (288 ± 4.64) (*N* = 3, 2-tailed unpaired t-test, *p* < 0.0001) **(g)**. Representative images showing Matrigel invasion assays using SKOV3 and HO-8910 cells with RPS6 knocked down and respective controls (magnification, 200×; scale bar: 200 μm) **(h)**. Representative graph of Matrigel invasion assay data for SKOV3 cells with RPS6 knocked down (15.33 ± 0.47 and 24.8 ± 4.95), compared with controls (shCon) (176 ± 20.05) (*N* = 3, 2-tailed unpaired t-test, *p* < 0.001) **(i)**. Representative graph of Matrigel invasion assay data for HO-8910 cells with RPS6 knocked down (26.7 ± 3.30 and 19.0 ± 7.93) compared with controls (shCon) (149.1 ± 22.70) (*N* = 3, 2-tailed unpaired t-test, *p* < 0.001) (J). Data are presented as mean ± SD for three independent experiments; ***p < 0.001, ****p < 0.0001

Scratch tests and transwell assays were performed to determine whether knockdown of RPS6 could suppress EOC cell migration and invasion. First, in scratch tests, the results demonstrated that knockdown of RPS6 reduced migration of SKOV3 (Fig. 4a, b) and HO-8910 (Fig. 4c, d) cells compared with the migration in the LV-shCon group at 24 h. At 48 h, knockdown of RPS6 more conspicuously inhibited cell migration ability (Fig. 4a-d). Subsequently, transwell assays were conducted to further assess the effect of RPS6 on cell migration. Following RPS6 knockdown, the number of cells transferred from the upper chamber to the base of the microporous membranes was markedly decreased relative to the shCon group for both SKOV3 and HO-8910 cell lines (Fig. 4e-g). Similarly, the invasion capacities of SKOV3 and HO-8910 cells were strongly reduced by RPS6 knockdown (Fig. 4h-j). These results indicate that RPS6 may facilitate the invasion and metastasis of ovarian cancer cells.

### Downregulation of RPS6 promotes cell cycle G0G1 phase arrest in EOC cells

**Fig. 5:**
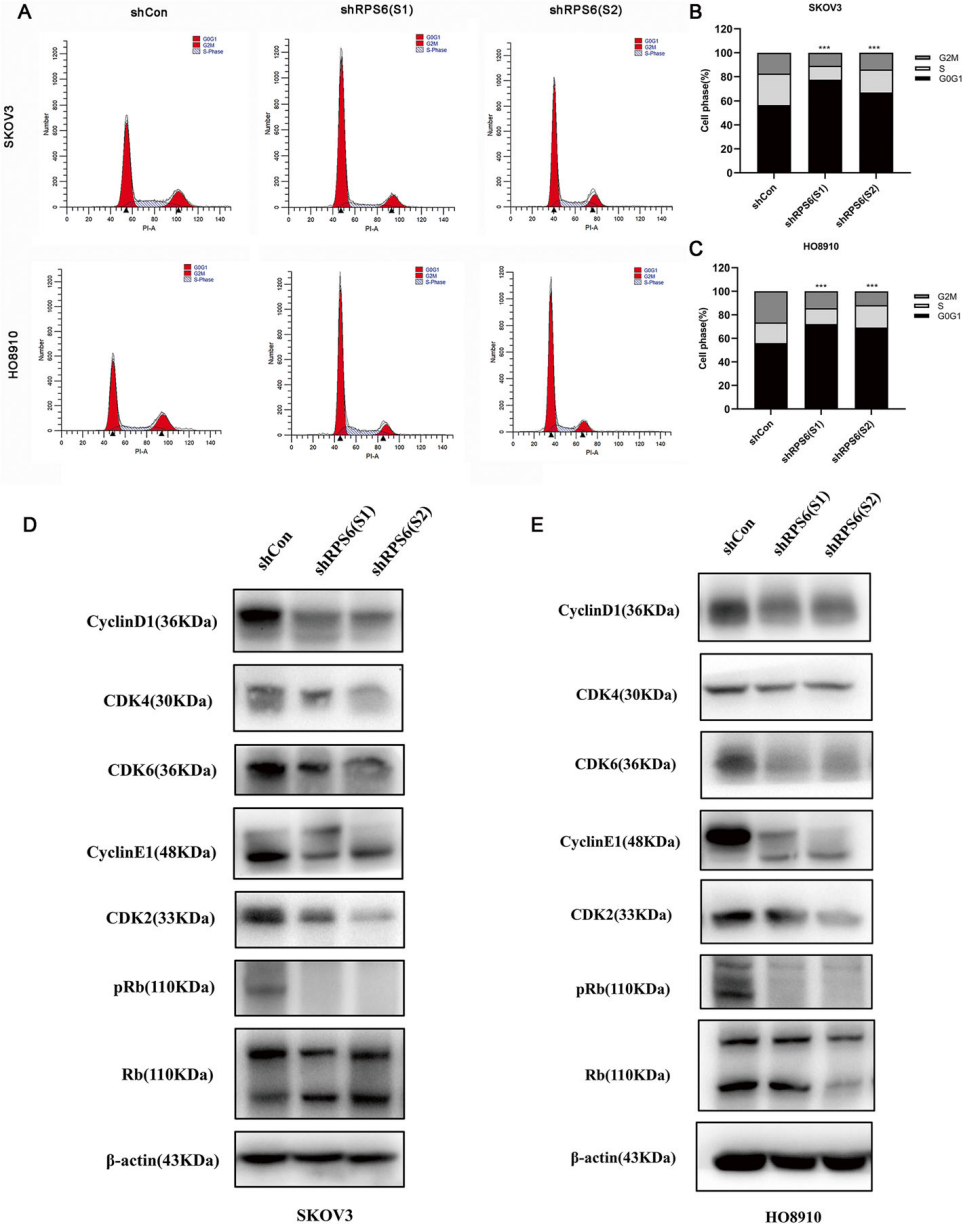
RPS6 knock down promotes cell cycle G1 phase arrest in ovarian cancer. SKOV3 and HO-8910 cell cycles were analyzed by flow cytometry. Representative images showing cell cycle arrest in G0G1 phase on RPS6 depletion are presented (**a**). Histogram illustrating the percentage of cells in each cell cycle phase. The results reveal that the proportion of cells in G0G1 phase increased, while the proportion in G2M phase decreased in SKOV3 and HO-8910 cells on RPS6 knockdown (**b** and **c**). Levels of cyclin D1, cyclin E, CDK2, CDK4, CDK6, and pRb were analyzed by western blotting following RPS6 knockdown in both SKOV3 and HO-8910 cells (**d** and **e**). All comparisons are with the LV-shCon control group; ***p < 0.001

To determine whether cell proliferation suppression by RPS6 knockdown resulted from alterations of the cell cycle, we conducted flow cytometry analysis. Interestingly, when RPS6 was knocked down, both SKOV3 and HO-8910 cells exhibited dramatic cell cycle arrest in the G0G1 phase, with reduced proportions of cells in the G2M phase (Fig. 5a). Regarding SKOV3 cells, percentage of cells in the G1 phase increased from 56.70% ± 0.60% (shCon) to 77.51% ± 0.38% (LV-shRPS6(S1) and 67.14% ± 0.25% (LV-shRPS6(S2) (Fig. 5b). Regarding HO8910 cells, the percentage of cells in the G1 phase increased from 56.04% ± 0.79% (shCon) to 72.23% ± 2.60% (LV-shRPS6(S1) and 69.28% ± 0.61% (LV-shRPS6(S2) (Fig. 5c).

To investigate the potential mechanism underlying these changes in the cell cycle, regulators critical for the G0/G1 checkpoint, including cyclin D1, cyclin E, CDK2, CDK4, CDK6, and phosphorylated retinoblastoma (pRb), were evaluated. Cells transiting from G0/G1 to S phase must pass a restriction point, mediated by the activities of Cyclin D-CDK4/6 and Cyclin E-CDK2 complexes, which are responsible for phosphorylation of Rb. Hypophosphorylation of pRb inhibits cell proliferation. In the present study, knockdown of RPS6 downregulated the levels of cyclin D1, cyclin E, CDK2, CDK4, CDK6, and pRb, relative to the shCon group, in both SKOV3 and HO-8910 cells (Fig. 5d, e). These findings indicate that RPS6 knockdown can trigger cell cycle arrest at the G0G1 phase, which leads to inhibition of ovarian cancer cell proliferation.

## Discussion

RPS6 is an indispensable ribosomal RNA-binding protein, which has a vital role in tumor transformation and cell cycle arrest, as well as cell proliferation, migration, and invasion [19]. Moreover, RPS6 is upregulated in various human cancers relative to normal tissues [20]; however, the role of RPS6 in ovarian cancer has not been fully elucidated to date.

In this study, we demonstrated that RPS6 is significantly upregulated in ovarian cancer, and that its expression is significantly associated with clinical stage and pathological grade. WHO (2014) classified the ovarian tumors into two types: low-grade carcinoma and high-grade carcinoma [21], which exhibit significant differences in their mechanisms of development, morphology and immunohistochemistry results, molecular genetics, and chemotherapy response [22]. The results here support the opinion that different pathological grade of ovarian cancer is with different genetic backgrounds. Moreover, the OS rate of the group with high RPS6 expression was significantly shorter than that of the group with low RPS6 expression, indicating that RPS6 levels are negatively associated with survival in patients with ovarian cancer. This is the first reported evaluation of the relationship between RPS6 and EOC clinicopathological characteristics and prognosis.

To gain a deeper understanding of the biological properties of RPS6 in EOC, we investigated the effects of silencing RPS6 on the SKOV3 and HO-8910 cell cycle, as well as their proliferation, colony formation, migration, and invasion abilities. Knockdown of RPS6 inhibited ovarian cancer cell proliferation, and this effect was enhanced over time. Knockdown of RPS6 also inhibited the rate of cell colony formation and induced cell cycle arrest at the G0G1 phase. These findings are consistent with the results of Chen et al. in their study of lung cancer cells [23].

Tumor cell migration and invasion are essential for the promotion of cancer invasion from the primary site into adjacent and distant tissues, which is a primary cause of tumor recurrence. Related studies have shown that RPS6 may play an important role in the migration and invasion of malignant tumors. Chen et al. [13] overexpressed RPS6 in the lung cancer cell line HBE and found that the migration ability of the cells was enhanced and matrix metalloproteinase (MMP)-2 expression was upregulated. Further, knockdown of RPS6 in SK-MES-1 and H1650 lung cancer cells reduced their invasive ability and decreased MMP9 and MMP2 expression, suggesting that high levels of RPS6 may promote lung cancer metastasis. Moreover, Kim et al. [15] deleted RPS6 in esophageal squamous cell carcinoma TE8 cells and found that the invasion and migration ability of the knockdown group was decreased. However, the role of RPS6 in EOC cell invasion and migration has not previously been reported. In this study, we demonstrated that knockdown of RPS6 also significantly inhibits the migration and invasion of EOC cells.

In follow-up experiments, we explored the specific means by which RPS6 knockdown inhibits ovarian cancer cell proliferation. Flow cytometry analysis of the ovarian cancer cell cycle revealed that cells in the G0G1 phase were significantly increased in the LV-shRPS6 groups, while cells in the G2M phase were simultaneously decreased. These changes in the cell cycle may be the primary mechanism by which RPS6 influences cell growth. Further support for this conclusion was provided by analysis of cell cycle-related proteins. Cyclin E is an important regulator of cell transition from G1 to S phase, through activation of CDK2. Under abnormal conditions, Cyclin E overexpression causes continuous activation of CDK2 and hyperphosphorylation of Rb protein, leading to abnormal cell proliferation and tumor development [24, 25]. Upregulation of Cyclin E is closely related to tumor progression and poor prognosis in breast cancer. In addition, the Cyclin D1-CDK4/6 complex initiates or regulates early G1 progression by phosphorylation of downstream Rb. Excessive phosphorylation of Rb attenuates cell cycle inhibition, allowing cells to proliferate uncontrollably [26]. Especially, deregulation of the CDK4/6–cyclin-D/p16–Rb signaling pathway is commonly found in ovarian cancer. Mutations of the Rb occurred in approximately 10% of ovarian cancers, and hemizygous deletions at the RB locus was found in 24 to 52% of ovarian cancers, which phenomenon was more commonly seen in invasive high-grade tumors [27]. Cyclin D1 was overexpressed in 18% of serous epithelial ovarian cancer and associated with a more aggressive tumor phenotype and poor prognosis, whereas CDK2 was shown to be amplified in a relatively small proportion (6.4%) of ovarian tumors [28]. Moreover, abnormal expression of CDK4/6 was described in 14 to 16% of EOC patients [29]. In this study, RPS6 knockdown resulted in decreased Cyclin E, CDK2, Cyclin D1, CDK4, and CDK6 levels, and reduced Rb phosphorylation, thus hindering the transition from G0G1 to S phase, causing most cells to be blocked in G0G1 phase, thereby inhibiting ovarian cancer cell growth. In addition, hypophosphorylated Rb can inhibit cell proliferation and induce cell differentiation, and higher levels of differentiation are associated with reduced tumor metastasis [30]. Hence, these findings also explain the inhibition of tumor invasion and migration ability upon RPS6 knockdown. Taken together, our findings confirm that RPS6-mediated changes in cell growth are closely related to cell cycle regulation.

Our research here provides evidence that high expression of RPS6 is a molecule event in the development of ovarian cancer and may therefore be a novel and promising drug target for anticancer therapies. Consistent with our results, studies showed that RPS6 was overexpression in non-small cell lung cancer [13], esophagus squamous cell carcinoma [15], pancreatic neuroendocrine tumors [31] and sarcoma [32]. However, further study is needed to characterize the molecular mechanisms of RPS6 in cancers fully, and in vivo studies are required to confirm these results followed by consideration of human trials.

## Conclusions

In conclusion, our results indicate that RPS6 is upregulated in tissues from patients with EOC, exerting carcinogenic effects, and that it is a prognostic indicator of poor clinical outcomes. RPS6 knockdown may influences tumor cell proliferation, migration, and invasion, and induces cell cycle arrest. Therefore, RPS6 may represent a novel marker and/or therapeutic target for patients with ovarian cancer. The specific regulatory mechanism mediated by RPS6 in ovarian cancer will be the subject of further studies in the future.

## Data Availability

The data used and/or analysed during the current study are available from the corresponding author on reasonable request.
